# Patients’ experience of accessing hepatitis C treatment through the Myanmar national hepatitis C treatment program: a qualitative evaluation

**DOI:** 10.1186/s12913-023-10456-0

**Published:** 2024-01-16

**Authors:** Bridget Draper, Win Lei Yee, Anna Bowring, Win Naing, Khin Pyone Kyi, Hla Htay, Jessica Howell, Margaret Hellard, Alisa Pedrana

**Affiliations:** 1 Disease Elimination Program, Burnet Institute, Melbourne, Australia; 2 School of Population Health and Preventive Medicine, Monash University, Melbourne, Australia; 3 Burnet Institute Myanmar, Yangon, Myanmar; 4 Yangon Specialty Hospital, Yangon, Myanmar; 5 Myanmar Liver Foundation, Yangon, Myanmar; 6 St Vincent’s Hospital, Melbourne, Australia; 7 Department of Medicine, University of Melbourne, Melbourne, Australia; 8 Hepatitis Services, Department of Infectious Diseases Alfred Hospital, Melbourne, Australia; 9 Doherty Institute, Melbourne, Australia; 10 School of Population and Global Health, University of Melbourne, Melbourne, Australia; 11 Health Services Research and Implementation, Monash Partners, Melbourne, Australia

**Keywords:** Patient experience, Barriers, Enablers, Hepatitis C, Hospital, National program, Low and middle-income countries, Myanmar

## Abstract

**Background:**

Globally, 56.8 million people are living with hepatitis C and over three-quarters of those reside in low and middle-income countries (LMICs). Barriers and enablers to hepatitis C care among people who inject drugs in high-income countries are well documented. However, there is scant literature describing the patient experience in LMICs. Understanding the barriers and enablers to care from the patient perspective is important to inform service refinements to improve accessibility and acceptability of hepatitis C care.

**Methods:**

We conducted a qualitative evaluation of the patient experience of accessing the national hepatitis C program at eight hospital sites in Myanmar. Semi-structured interviews were conducted with four to five participants per site. Interview data were analysed thematically, with deductive codes from Levesque et al.’s (2013) Framework on patient-centred access to healthcare.

**Results:**

Across the eight sites, 38 participants who had completed treatment were interviewed. Barriers to accessing care were mostly related to attending for care and included travel time and costs, multiple appointments, and wait times. Some participants described how they did not receive adequate information on hepatitis C, particularly its transmission routes, and on the level of cirrhosis of their liver and what they were required to do after treatment (i.e. reduce alcohol consumption, liver cirrhosis monitoring). Many participants commented that they had few or no opportunities to ask questions. Provision of treatment at no cost was essential to accessibility, and gratitude for free treatment led to high acceptability of care, even when accessing care was inconvenient.

**Conclusions:**

These findings highlight the importance of streamlining and decentralising health services, adequate human resourcing and training, and affordable treatment in maximising the accessibility and acceptability of hepatitis C care in LMICs. Findings from this work will inform future service delivery refinements for national program and other decentralised programs to improve accessibility and acceptability of hepatitis C care in Myanmar.

**Supplementary Information:**

The online version contains supplementary material available at 10.1186/s12913-023-10456-0.

## Background

Globally, approximately 56.8 million people are living with hepatitis C and over three-quarters of those reside in low and middle-income countries (LMICs) [1]. Since the introduction of direct-acting antiviral (DAA) treatments for hepatitis C, there has been a rapid increase in the uptake of treatment globally [2]. However, many LMICs still face a multitude of barriers to scaling up hepatitis C treatment [3]. Key barriers to scale-up are inadequate specialist workforces and the high cost of DAAs and diagnostics [3, 4]. Global guidance supports task-shifting of treatment to non-specialist prescribers [5]. Task-shifting from specialist prescribers (e.g., hepatologists, gastroenterologists or infectious diseases physicians) to non-specialist prescribers would expand access to treatment, and ensure access to specialists for those with advanced liver disease [6]. In many settings, the number of people able to enrol in low-cost or free public hepatitis C treatment programs is capped. The alternative is to access hepatitis C treatment via private providers, but this means the cost of DAAs and consultations is unaffordable to most people [7].

In an effort to increase uptake of hepatitis C testing and treatment and improve retention through the care cascade, services should be accessible and acceptable to people living with hepatitis C [8]. Globally, a key barrier to increasing treatment uptake in LMICs is diagnosis of current infection [7]. Typically, people are tested for hepatitis C antibodies to determine past exposure, but do not receive an RNA test to confirm active infection and are not linked to treatment [9]. Often testing and treatment are provided in separate locations and require multiple appointments to complete the diagnostic pathway and initiate treatment [9, 10]. Evaluating the accessibility and acceptability of available programs is crucial to understand the barriers and enablers to accessing care; understanding these barriers and enablers at a country-level will help inform the national response. Overcoming these barriers will likely enable more people to initiate and complete treatment [11].

To date, much of the literature on hepatitis C testing and treatment is from high-income countries and focuses on people who inject drugs [11, 12], who are disproportionately affected by hepatitis C in these settings [13, 14]. A recent review examined the barriers and enablers to hepatitis C care among marginalised groups (predominately people who inject drugs) in high-income countries, and identified patient-level and health-system level factors that influenced treatment initiation [11]. Patient-level barriers included lack of urgency to start treatment due to asymptomatic infection, competing life priorities (e.g. housing, active drug use), concerns about side effects, and fear of experiencing stigma when seeking healthcare [11]. Aside from this review, other literature has cited the cost of testing and treatment, and transportation issues (i.e. availability, cost) as barriers to accessing care [15]. In addition, from the provider perspective, barriers to providing hepatitis C care can include lack of appropriate training and support, heavy workloads, short visit times, and their own reticence to treat people with complex socio-economic situations and mental health comorbidities [11, 15, 16]. Health system barriers predominately related to gaps in continuity of care and policies requiring abstinence from drug use [11]. Enablers of care identified by the review included having a trustworthy provider and a multidisciplinary team able to address health and social issues other than hepatitis C [11].

The few studies describing barriers and enablers to care in LMICs report many of the same barriers as the high-income country literature; some of the LMIC studies have focused on people who inject drugs [11, 17, 18]. For example, studies conducted in Rwanda of the general population accessing care and South Africa of people who inject drugs found that key barriers to accessing hepatitis C care were the cost of testing and treatment, and travel distances and related costs [17, 18]. Another barrier reported in these studies was extensive wait times to access hospital-based care for hepatitis C [17, 18]; this is not commonly reported in high-income country studies. Overcoming these barriers required considerable effort from the patient to successfully complete the full care pathway [17]. In addition, many people who inject drugs interviewed in South Africa stated they did not attend because they forgot the appointment date or were not motivated to access treatment, either due to perceived lack of urgency or fatalism about dying due to hepatitis C or from injecting drug use-related harms [18]. Recent qualitative work from Vietnam outlined the various barriers to seeking care for viral hepatitis, including limited and/or incorrect understanding of how they acquired hepatitis C, lack of trust in the local healthcare system to provide appropriate treatment, difficulty reaching care in centralised hospitals, challenges navigating insurance schemes to cover treatment, and cost of testing and treatment [19]. In addition, recent qualitative work assessing the acceptability of a community-based model of care in Yangon, Myanmar found that flexible appointment scheduling, short wait times and rapid return of results increased the clinic’s accessibility [20].

In Myanmar, an LMIC in South-East Asia, over one million people are living with hepatitis C. Population prevalence of hepatitis C antibody is 2.7%, with antibody prevalence among people who inject drugs at 57% [21, 22]. A prevalence survey found that hepatitis C antibody positivity was higher among older age groups, specifically 50–59 years, and was associated with previous blood transfusion, indicating exposure prior to blood donor screening measures were introduced in 2000 [21]. The Myanmar National Hepatitis Control Program (NHCP) released the first National Strategic Plan [23] and National Action Plan (2016–2020) [24] in 2016, outlining targets for viral hepatitis testing and treatment for 2030. For hepatitis C, targets included diagnosing 50% and treating 50% of people diagnosed with current hepatitis C infection by 2030 [24]. Following the release of the national treatment guidelines in 2017 [25], the NHCP launched the first phase of the national treatment program at eight sites in Yangon, Mandalay, and Naypyidaw [26]. This first phase, referred to as the QuickStart Program, provided approximately 2000 treatment courses at no cost to people in the program in the first year. Following this first phase, the program was expanded to more than 13 sites and additional treatment capacity made available through a public–private partnership option, whereby people could pay for subsidised pre-treatment investigations and DAAs and are treated at the same hospital sites. Across Myanmar, hepatitis C testing and treatment is also available through various private clinics and non-government-organisation-run clinics, or through research projects [24, 27], albeit often only in major cities or in Kachin State, where the prevalence of hepatitis C among people who inject drugs is disproportionately high [28].

Here we describe the barriers and enablers to accessing hepatitis C care through the first phase of the national QuickStart program in Myanmar and patient satisfaction with the care received.

## Methods

### Study setting

Within the first phase of implementation from 2017 to 2018, the national program treated people living with both mono-infection (1200) and hepatitis C/HIV co-infection (800). People living with hepatitis C attended the hospital sites (study sites) through self-referral or doctor referral to hospital site, with people then enrolled in the national program in order of presentation to the site and some sites prioritising those with cirrhosis. There were eight district-level public hospital sites located in Yangon, Mandalay, and Naypyidaw; two were infectious diseases hospitals and six were general hospitals. These sites provided hepatitis C RNA testing and DAA treatment in the outpatient department at no cost to those enrolled. However, some of these sites required people to pay for pre-treatment evaluation investigations, including liver function tests and chest X-rays (costing USD0.50–4.00 per investigation). Implementation of this national program was funded from the domestic budget, and received technical support from the Clinton Health Access Initiative (CHAI).

From enrolment to initiating DAAs, people typically attended the hospital site for various consultations with doctors (generally, either hepatologists or infectious diseases physicians) and investigations on two to five occasions. Consultations, investigations (e.g. chest X-ray to exclude tuberculosis infection, ultrasound for cirrhosis assessment), phlebotomy for blood tests (including for hepatitis C RNA testing, even if blood samples were then transported off-site for blood tests to be performed at an external laboratory), and DAA dispensing all occurred at the district hospital, within different wards. Specific clinical pathways differed slightly by site. For example, some sites required people to collect their own test results prior to their next consultation with the doctor and some sites implemented on-treatment monitoring of renal and liver function.

### Study design

The NHCP commissioned the Burnet Institute and CHAI to evaluate the qualitative and quantitative components, respectively, of the first phase of program implementation. CHAI has published aspects of the quantitative evaluation findings elsewhere [29]. The qualitative component involved semi-structured interviews with a subset of staff (doctors, nurses, laboratory technicians and data collectors) and people enrolled in the treatment program from each site. Data from interviews with staff are not included in these analyses and will be analysed and published elsewhere.

### Study participants

People enrolled in the program were eligible to participate in the qualitative sub-study on patient acceptability if they were aged 18 years and over, had received hepatitis C treatment within the national program, were due for their SVR result return appointment, and were willing and able to consent to the qualitative sub-study.

A convenience sample of people attending for the appointment to receive their treatment outcome results – an RNA test to confirm sustained virological response (SVR) 12 weeks after treatment completion – were selected for participation in the qualitative sub-study for this evaluation. Hospital staff approached people with appointments scheduled for the assigned research interview days and invited them to participate, and scheduled appointments with research staff for interested potential participants. Across the eight sites, 38 participants were recruited between August and September 2018.

### Study procedures

#### Data collection

Three research staff from the Burnet Institute, trained in qualitative interviewing, conducted interviews in Burmese (WLY, SMT, AAM). Interviews took place in private rooms at the hospital sites. Participants did not receive any incentives for participating in the interviews. A semi-structured interview guide was developed to explore the participants’ journey through hepatitis C care, including diagnosis and linkage to care at hospital sites, how people found out about the national program and how to access it, the wait time for consultations and investigations, whether they needed to pay for any aspects of their care, the transport requirements for attending, and their satisfaction with the care received (see Supplementary Material 1. Participant Interview Guide).

Interviews took 40–70 min and were audio-recorded. They were transcribed in Burmese using denaturalised transcription (focus on informational content) and then translated into English by professional transcribers and translators contracted by Burnet Institute. Burmese transcripts and English translations were checked by Burnet Institute medical staff with technical expertise in hepatitis C for accuracy, with a focus on topic specific terms.

#### Data analysis

Another researcher (BLD) managed and analysed the English interview data using NVivo 20 (QSR International), including performing initial coding. The researcher then used the visual diagram web platform Lucidchart (Lucid Software Inc) to complete iterative coding and interpretation, using a flexible tree diagram with additional text to iteratively organise the themes [30]. The iterative coding and interpretation technique refers to iteratively coding and organising emergent themes within a coding framework to interpret and report on findings, guided by the research questions which create deductive codes and additional emergent inductive codes [30].

Interview data from participants were analysed thematically, with deductive codes from the Levesque Framework [31], supported by iterative coding techniques [30]. The Levesque Framework [31] on patient-centred access to healthcare defines access as the opportunity to identify healthcare needs, seek healthcare services, to reach services, to obtain/use services, and to have needs for services fulfilled. The five dimensions of health system/service accessibility are Approachability, Acceptability, Availability and accommodation, Affordability, and Appropriateness. Five corresponding abilities of population to interact with these dimensions of accessibility, to generate ‘access’, are Ability to perceive, Ability to seek, Ability to reach, Ability to pay, and Ability to engage.

Results are presented with reference to the Levesque Framework, with barriers and enablers presented together within each domain. Inductive themes related to how accessibility of care is viewed and influenced by other factors are summarized separately. Preliminary findings and interpretations were validated with Myanmar National Hepatitis Control Program senior staff, senior hepatologists and key partners via dissemination workshops where stakeholders had the opportunity to further explain context of findings to enhance understanding and interpretation.

It is worth noting that the translation of quotes from Myanmar language to English can result in removal of the context or implied meaning of the quote; the interpretation of these translated quotes have been cross-checked with Myanmar research team to ensure validity of translation and interpretation with the original interview transcripts and/or audio.

#### Ethics

Retrospective ethics approval of the program evaluation and publication of its findings to a wider audience were obtained from the Myanmar University of Public Health Institutional Review Board (Project: 2020/3) and the Alfred Health Human Research Ethics Committee (Project: #205/20), respectively. Participants provided written informed consent prior to interviews.

## Results

### Participant characteristics

Participant characteristics are summarised in Table 1. Of the 38 participants interviewed, just over half were male, and median age was 47 years (Table 1). The most commonly reported risk factor for hepatitis C acquisition was unsafe healthcare procedures, including blood transfusions, injections from local clinics, and dental work. One fifth of participants reported a history of injecting drug use. Most participants (n = 36) had received their treatment outcome result at the time of interview, of whom 34 had achieved SVR and two had not achieved SVR.

**Table 1:** Participant demographics

	N = 38 n (%)
Age (median, IQR)	47 (IQR: 39.5, 55.75) (range: 28–70)
Sex	
Male	20 (53)
Female	18 (47)
Reported hepatitis C risk factors*	
Potentially unsafe healthcare procedures	25 (66)
Family member with hepatitis C	15 (39)
Injecting drug use	8 (21)
Unknown	1 (3)
Treatment outcome (self-reported)	
Achieved Sustained Virological Response	34 (90)
Did not achieve Sustained Virological Response	2 (5)
No result reported at time of interview	2 (5)

*Participants were asked about any possible risk factors; if reporting multiple risk factors, these responses are recorded in each category. Percentages will not add up to 100%. One participant did not report any potential risk factors, classified as “Unknown”

### Barriers and enablers

**Fig. 1:**
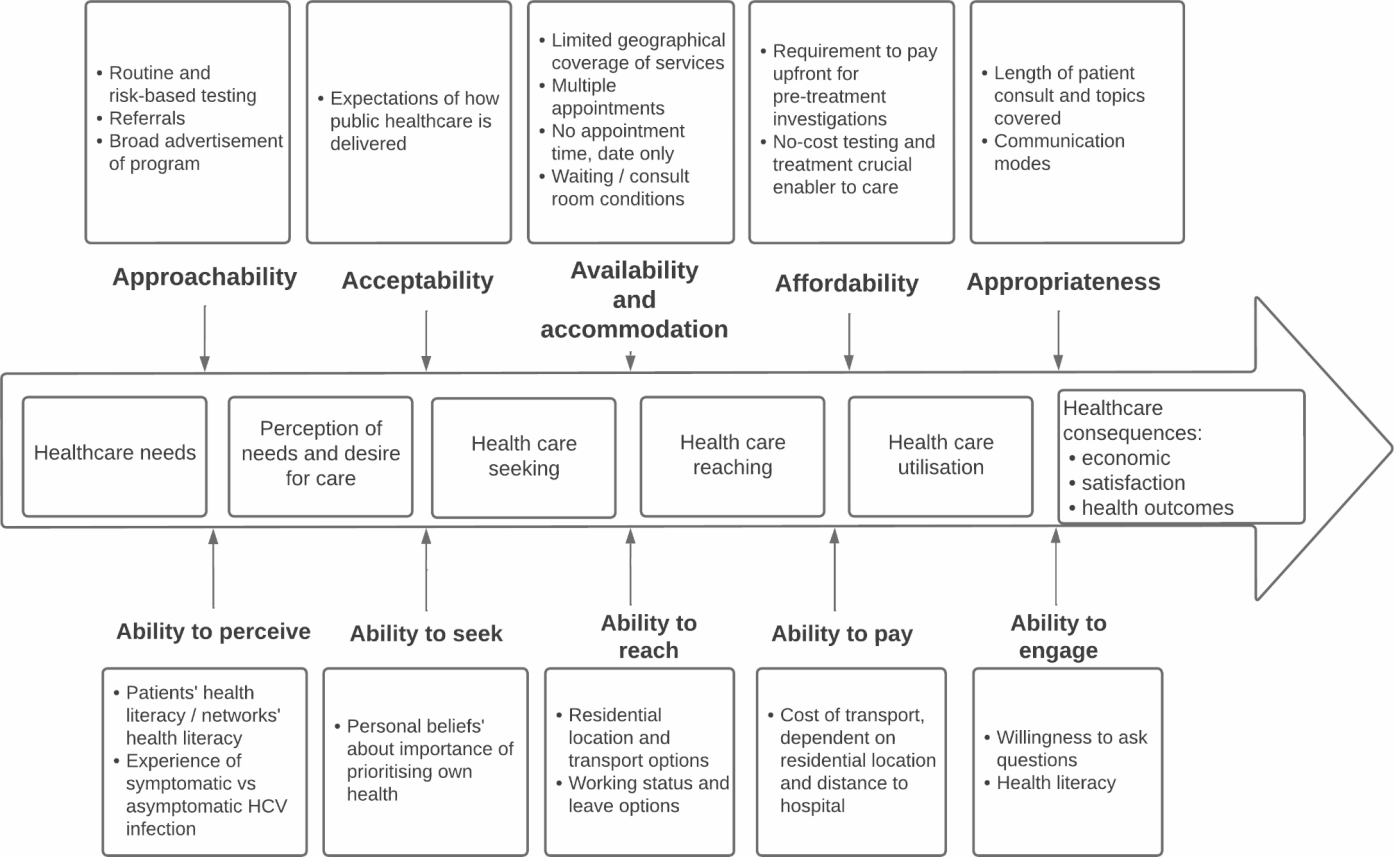
Determinants of accessing care, guided by [31] framework

These themes are summarised in Table 2 and presented in an adapted schematic of the Levesque Framework domains in Fig. 1. Overall, the themes identified were similar across all sites, with the key differences by site related to whether participants were required to pay for any investigations and how pre-treatment education was delivered.

**Table 2:** Summary of potential barriers and enablers to accessing care in Myanmar QuickStart hepatitis C treatment program (Phase I)

Themes	Potential Barriers to Accessing Care	Enablers to Accessing Care
Approachability	Not reported (N/R)	Routine and risk-based hepatitis C testing
		Referral by local doctor to NHCP site
		Advertisement of program, including free treatment
Ability to perceive	Experiencing asymptomatic infection	Experiencing liver-related symptoms
		Family and friends’ knowledge about the availability and benefits of hepatitis C treatment
Acceptability	N/R	Expectations of how public healthcare is delivered
Ability to seek	N/R	Personal beliefs’ about importance of prioritising own health
Availability and Accommodation	Limited geographical coverage	N/R
	Requirement to attend on multiple occasions, within one week at times	
	Lack of appointment time booking (only date booked) contributed to long wait times for consultations	
	Lack of privacy	
	Insufficient seating, crowding or noisy waiting room	
Ability to reach	If living outside of city, time and means to travel to hospital	Cheap and efficient transport if living within city
	Casual workers lost income to attend appointments	Paid medical leave to attend appointments
Affordability	Requirement to pay upfront for pre-treatment investigations	Provision of no-cost testing and treatment was crucial enabler
Ability to pay	If living outside of city, transport costs were high	If living in the city, costs of transport were generally low depending on mode and distance; public transport was cheaper than other modes
Appropriateness	Patient education solely focused on DAA adherence only Short consultations	Adequate length of consult to allow for discussion of hepatitis C, the treatment plan and liver health; including individual health status
		Written materials that cover key topics of interest, including transmission routes, liver health, reducing risk of re-infection, ongoing Ab positive status.
		Kind, patient, and friendly staff
	Wait time was described as long but was not identified as a barrier to accessing care	Well-implemented reminder and booking system
Ability to engage	Unwillingness to ask questions due to: - limited opportunities to ask questions in short consult times / consults in groups of ten - power imbalance / cultural norms influencing comfort in asking questions of doctors - reluctance to bother doctors and nurses as they were very busy - internalised shame, typically among those who reported inject drug use or men who had sex with men	When people enrolled in the program did ask questions, they received clear responses with useful information
	Limited health literacy on hepatitis C and liver health	

N/R = not reported

### Approachability and ability to perceive

This theme relates the participants’ ability to identify their need to access hepatitis C treatment and the approachability of the service, including the availability of service information and referral pathways that enabled linkage to care.

#### Test and refer pathways

Most participants were diagnosed with hepatitis C through routine or risk-based testing offered by their local doctor or hospital service. For example, many participants were diagnosed when they were donating blood, prior to surgery, or regular testing as part of an HIV treatment program.

“They regularly do blood tests every six months because I have HIV. From that, I know I have [hepatitis] C.” – QuickStart Participant #13 (QS-13)

Proactive offering of testing at these healthcare encounters was an enabler for perceiving the need for hepatitis C care; this is particularly important given the asymptomatic nature of hepatitis C infection, which some participants noted as a factor delaying their care-seeking.

#### Family and friends’ knowledge about Hepatitis C

Many participants were diagnosed many years ago. For these participants, experiencing symptoms they believed were due to hepatitis C and encouragement from family or friends to seek treatment through the new NHCP program were enablers to perceiving the need for treatment.

“I didn’t feel good, [I] felt dizzy with fatigue. And I hadn’t gone for follow-up for hepatitis C for a long time. So, my daughter said that there is a 1000 bedded hospital near to her home and told me to go and have [a] consultation.” - QS-21

#### Advertisement of the treatment program

Some participants described how they found out about the availability of treatment at NHCP sites directly or indirectly from friends or family through Facebook or mainstream media surrounding the launch of the program; wide advertisement of the program and availability of treatments increased approachability of care and was an enabler to accessing care.

“My wife’s sister found out the information on Facebook that the hospitals were providing treatments for hepatitis C.” – QS-16

After diagnosis, some participants were then referred to a NHCP site for registration for the treatment program, either through written referral in their patient medical record booklet or verbal advice to attend a local NHCP site where they were later offered treatment through the program.

“He wrote a referral note in my medical record book, and referred me to the Liver Unit in this hospital… When I got here and showed this book, [the] doctors from this hospital welcomed me.” – QS-18

### Acceptability and ability to seek

#### Personal beliefs about importance of prioritising health

Most participants described how it was important for them to prioritise their own health and to seek out treatment to cure their hepatitis C. This prioritisation of health was framed as the underlying enabler for overcoming any barriers to accessing care and as a motivation to access care, particularly as the treatment was provided at no cost in this program. For example, when asked if satisfied with the number of visits required, one patient described their motivation to attend for care in this program was to take care of their own health by accessing free treatment. At the same time as recognising the value of this program, they described the difficulties accessing care at the busy clinic.

They replied:

“I must come because of my health. There is nothing to be unsatisfied [about]… How should I say? This is a free program, and the doctors and nurses are very busy, there are a lot of patients whom they need to take care [of]. I have satisfaction.” – QS-09

#### Expectations of the public health system

Some participants described how they arrived early at the hospital (6–7 am) and queued for consultations (by placing their patient medical book in the pile at reception), waiting between three and six hours generally, and were seen in order of arrival. Other participants arrived 8–9 am and were seen by 12 noon; wait times varied by site and appointment type (e.g. initial consultation, phlebotomy, DAA dispensing). Given there is no time component to appointments (people are only told what date/day of week to attend), wait times varied depending on when people arrived and how many people were in the queue before them.

“Although I got here at 6 am, about 30 books were already there. I had to line-up to wait [until] 12:30pm” - QS-18

Some participants travelling from outside urban centres described how they had to arrive at 9 am to have blood samples taken to ensure their sample could be couriered to the external laboratory for confirmatory hepatitis C testing; one patient described how she arrived at 11 am from outside Yangon and missed the appointment window for phlebotomy.

“One or two times, I had to go back without getting blood tests because I arrived late and could not catch the delivery to NHL [National Health Laboratory].” - QS-32

Many participants described how they accepted the need to arrive early to queue and long wait times to be able to access free healthcare, understanding that that was how the public healthcare system functioned and accepting that it was a time-consuming process. As illustrated above, this acceptance of the care model was influenced by being unable to afford to access treatment elsewhere.

“No issue for me – [the] costs are inexpensive comparing to that of private clinics. I had to wait with patience for my healthcare … I am not a boss and could not go to specialists to take treatments and thus I had to wait some time with patience…” – QS-35

However, insufficient seating, crowding or noisy waiting rooms made the wait time uncomfortable for some participants.

“Sometimes, my legs became frozen due to standing while waiting as there was no chair [for me] to sit.” - QS-36

### Availability and accommodation and ability to reach

#### Limited geographical coverage

Poor geographical coverage of hepatitis C treatment program sites, meaning that the location of the hospital site was far from where the patient lived, was a barrier to accessing care. For those who lived within one hour of the hospital, most remarked that they did not encounter any difficulties in reaching the site. Conversely, as described above, many participants from outside major urban centres had to travel extensively to reach the nearest hospital site. For some participants, travelling took over two hours and multiple forms of transport, with associated costs.

“I come by motorboat. After that I take a taxi. It takes around two hours” – QS-09

“If I come by car at three o’clock in the morning, I can arrive here at seven o’clock in the morning. It costs around forty to fifty thousand kyats [USD22-28]. So we try not to sleep in Yangon to reduce accommodation charge.” – QS-32

The distance travelled to reach the hospital affected when people were able to arrive to queue for care; people who travelled further often arrived later and therefore had to wait longer to see the doctor. People who had to travel from outside the cities where the hospitals are located made major sacrifices to arrive on time and then to return home within the same day, with the double burden of having waited longer to see a doctor that day.

For example, a participant who left Maw Kyun in Ayerwaddy at 3 am to arrive in Yangon at 7 am commented that they had to leave this early to see the doctor and attend phlebotomy appointments on time, to ensure their blood sample was included in those couriered to the external laboratory on the same day.

#### Lost income for ‘daily wagers’

For participants who worked casually (‘daily wagers’), most described how they were able to take the day off work or arrange for someone to cover for them at their shop, but that it often resulted in lost income.

“Convenient? We are the daily wagers [casual workers]. But I came on the appointed dates regularly … I came to the clinic on time regularly no matter how difficult it was.” – QS-25

Others were entitled to take paid medical leave, but often experienced some pressure to complete their work in fewer hours instead. Having sufficient work flexibility was an enabler of access to care, but the spectre of lost income for casual workers or small business owners was clearly a barrier to accessing care for many participants in Myanmar.

#### Requirement to attend on multiple occasions

The requirement to attend for appointments on multiple occasions, often within one week, was another barrier to care, with participants having to attend up to four times across two weeks to complete the tasks required to initiate DAA treatment. This requirement varied by site, and the extent to which this was a barrier depended on the distance from the patient’s home to the service and work and other commitments.

“Blood collection was done on Monday, results were collected on Tuesday and [I] had to re-visit here again on Wednesday.” – QS-15

When asked if they would prefer to receive the results on the same day, rather than waiting two weeks to receive them, one participant replied:

“Generally, it would be good to get the results on the same day. Because if we have to come back on the next day, we need to cancel work and may have some inconveniences.” – QS-29

#### Lack of privacy

At most sites, there were too few consultation rooms to accommodate the patient workload. Some participants described the lack of privacy in consultations and how that reduced the ability to have individualised care. For most participants interviewed, the lack of privacy was not framed as a major barrier to accessing care. Most participants referred to the fact that all the people waiting had the same disease, implying there was no risk of stigma or discrimination and a sense of safety in this setting. However, only one fifth of participants interviewed reported injecting drug use as a risk factor (even fewer reported current injecting drug use); stigma relating to injecting drug use may increase the significance of lack of privacy among these participants.

“I don’t feel anything because all the patients from this liver unit have the same disease and we can talk openly. There’s no consequence if the others hear.” – QS-04

### Affordability and ability to pay

#### Crucial importance of free treatment

Participants viewed the lack of cost as extremely important to access to care; it was a major driver of the decision to access treatment through the national program. Participants described how they had previously delayed seeking hepatitis C treatment due to the unaffordable cost.

“I could not afford to take treatments which might cost about forty to fifty million kyats [USD22500–28100].” – QS-36

“To take treatments by myself and own my own business, it would not be easy to save up the money to take treatments. Frankly saying, I am not sure whether I might be able to take treatments after three to five years or not.” – QS-21

#### Cost of pre-treatment investigations & pre-treatment supplements

At some sites, people were required to pay for pre-treatment investigations such as liver function tests, ultrasounds, and X-rays. In some instances, people with hepatitis C and HIV co-infection were reimbursed by the HIV program. The costs generally ranged from 1000 to 7000 kyats (USD0.50–4).

“The costs [of investigations] were more than four thousand kyats and seven thousand kyats [USD2-4]. I had to pay for every blood test done. One nurse took blood out and [I] had to pay money and give [pathology request] form to another nurse.” – QS-15

The interviewed participants managed to cover these costs as they continued their treatment. This participant commented on how the costs were affordable:

“These costs were not much … Starting from one thousand to two thousand kyats [USD0.50-1]… some tests were more costly, and total was then more than ten thousand kyats[USD5.60].” – QS-18

It is unclear whether participants had to borrow money from family or friends, make sacrifices or defer other expenses to afford these costs. However, these costs may prevent others living with hepatitis C from successfully accessing care; as the participants themselves posited.

A few participants commented that they were prescribed liver health supplements to take while they waited to start DAA treatment by the national program doctors. Most received one month free and then had to buy extra months themselves (the cost of these supplements was not reported within the interviews).

#### Travel costs

Travel costs were low for most participants, who commented that they were not difficult to cover, with most participants’ transport costs ranging from 200 to 10,000 kyats (USD0.10–5.60) per visit depending on the mode and distance, with most reporting costs of under 1000 kyats (USD0.50) for a return trip.

For one participant, the cost of return transport and lunch totalled 5,000 kyats (USD2.80). When asked about the cost of attending, they said:

“Around four thousand to five thousand – that’s including lunch. For cost, it is not difficult for me, it is not much.” - QS-04

Participants who needed to travel further reported higher travel costs, ranging from 40,000 to 60,000 kyats (USD22–39).

“It costs a lot, about fifty thousand without accommodation cost.” – QS-21

Minimum wage in Myanmar was 4800 kyats a day in 2018 (approx. USD3.50 at 2018 rates) [32], meaning that the accommodation costs quoted above are more than 10 days of income for low wage earners.

### Appropriateness and ability to Engage

This section describes barriers and enablers to providing appropriate care and the corresponding ability of people to engage with care, and the links between them and satisfaction with overall services and health outcomes.

#### Willingness to ask questions

Some participants did ask questions and receive adequate responses; this is described further below in the context of pre-treatment education. However, typically participants reported that they did not ask any questions of doctors and nurses.

The main reasons for not asking questions were related to structural lack of opportunities, either due to having short consultation times, or the pre-treatment education being conducted in groups of 10 people. Many participants commented that they did not want to bother the busy staff.

This issue was compounded by the power imbalance between patients and doctors and by cultural norms that govern personal interactions, with politeness being a central factor.

“Because there are a lot of patients. I don’t want to disrupt because of me. That’s why I would not ask any questions.” – QS-03

In addition, this power imbalance was likely exacerbated by peoples’ inability to afford treatment elsewhere, hence seeking care within the public health system and receiving free treatment; with some participants commenting that they would expect better care if seeking care at a private clinic.

Some participants did not ask questions due to internalised shame around how they acquired hepatitis C. For example, when asked why they did not ask for clarifications on topics they did not understand, one participant responded:

“Because I was the one who made mistakes. I lived my life badly. I was on drugs… having sex with the same and opposite sex. ” – QS-10

Participants commented that they would like to know more about their own individual situation, particularly about their liver disease status and how to avoid reinfection.

“I would like to get information on how to behave and what should I do for my disease … I want to know more details about my situation.” – QS-09

#### Content of pre-treatment education

Many participants were unsatisfied with the content covered in the consultations, the lack of written resources, and the short time spent explaining disease transmission routes and the long-term management plan for their liver health. The participants’ descriptions of pre-treatment education suggest doctors and nurses focused heavily on DAA adherence, with less attention to side effects, broader liver health care, transmission routes, and reinfection risk reduction.

“‘You have hepatitis C. You need to take hepatitis C treatment regularly and you must not drink alcohol.’ That’s all … I only understood on what time I should take the medicines.” – QS-08

Receiving minimal information reduced participants’ satisfaction of care. Many participants wanted to know about transmission routes to understand how they might have acquired hepatitis C and how to avoid reinfection, particularly among those who went so far as to avoid sharing saliva with family or friends through bringing their own cups and eating utensils to guard against onward transmission of hepatitis C. Also, participants wanted to know more about their individual liver health situation, and what they needed to do for their liver after finishing treatment. In addition, a few participants experienced unexpected severe side effects of treatment that reduced their ability to cope with daily life, including paid work and household tasks.

“It would be much better if each and every patient got all the necessary information thoroughly but it could not happen due to crowded patients and less time for health care staff.” – QS-17

Nonetheless, for some participants, the focus on DAAs and importance of adherence was enough; these participants trusted the doctors were providing them with appropriate treatment, and that all they needed to know in order to get cured was how and when to take the treatment.

Participants from some sites reported receiving written information and explanations about common side effects and information about liver disease in general.

“The nurses explained. They gave us an information sheet about how to take medicine, how to prevent spreading [hepatitis C] to others … They told us in detail.” – QS-13

“The doctors and nurses explained [it to] the patients until they understood. They helped us with great care and passion.” – QS-24

According to participants’ recollections, some doctors and nurses provided incorrect information about common transmission routes to people, confirming misconceptions in the community surrounding transmission through saliva shared via eating and drinking utensils. For example, some participants said they were told that hepatitis C transmission is linked to eating certain foods.

“They told me that [hepatitis C] can be transmitted from eating barbeque.” – QS-01

However, it is unclear whether these participants were confusing hepatitis viruses. The NHCP provides written resources on all hepatitis viruses for distribution at hospitals.

“[The Consultant] said I don’t need to avoid anything but not to eat roots and eggs which can cause hepatitis … He said it is alright for hepatitis C but if I am infected with hepatitis B, I need to avoid many things. It can be transmitted through living together with the children and disposing the saliva, etc.” – QS-22

Experiences of patient education varied between sites, with experiences from some sites demonstrating that it was provided well. Doctors and nurses involved in delivering the program underwent a standardised clinical training course as part of the national roll-out.

For example, at some sites, many participants described how they had enough time with doctors and nurses to be able to ask questions and receive detailed answers. They described receiving clear explanations about hepatitis C, the treatment plan, liver health, and ongoing antibody positivity post-cure. There was a strong preference for clear health information messages in simple language and for opportunities to ask questions; the type of the provider (doctor or nurse) delivering this information was less important. Explanations were often accompanied by written information pamphlets or the certificate of cure that can be used to communicate ongoing antibody positivity status to workplaces or other health facilities.

“I asked the nurses and doctors if I didn’t understand anything, and they explained it to me. I hardly needed to ask them again because they explained it to me very simply and clearly.” – QS-34

#### Kind and considerate staff

Overwhelmingly, participants described the staff as kind, patient, and friendly. The participants felt that the staff had their best interests at heart and appreciated the kindness shown towards them.

“All the doctors and nurses were kind.” – QS-12

“The nurses took really good care of me with kind hearts.” – QS-07

“They showed me the locations, the counters for queuing up the medical books, and guided me on what to do where.” – QS-19

Participants universally commented on how useful they found it when the nurses called to remind them about their upcoming appointments or to remind them to pick up their DAAs prior to dispensing.

#### Satisfaction with care received

When asked, almost all participants reported being satisfied with the care they received. Participants felt that the staff were friendly and kind, their information was kept securely, the facilities were clean, and the staff did not ask them to come for appointments unnecessarily. Participants believed that the test results were accurate and the treatment regimen chosen was appropriate; almost all participants were satisfied with the services received. Gratitude for no-cost treatment was a major contributor to satisfaction with care.

“I felt satisfied for all the services. I felt thankful.” – QS-07

A few participants had specific complaints about the level of cleanliness of the facilities or rude staff. For example, one participant who injected drugs felt that the staff treated the people enrolled in the program differently because they were providing treatment at no cost:

“Since they are giving the treatment free of charge … they were not that friendly … It was like they are giving these drugs freely and we have to follow whatever they said. Like this.” – QS-08

On a few occasions, participants commented that they sought confirmation of cirrhosis status or other co-morbidities at private clinics, and that this was often suggested by the treating doctor. Others commented how they could not complain because they were attending for free care, but would expect better care if they were paying for it at a private clinic.

## Discussion

The findings of this study describe the experiences of people enrolled in the NHCP hepatitis C QuickStart treatment program in Myanmar in its first phase of implementation. We identified barriers and enablers to accessing care among a subset of people who completed treatment. Barriers identified within this study did not prevent the participants interviewed from accessing care, but were identified as challenges to accessing care and may be barriers to others seeking hepatitis C treatment in Myanmar.

While many participants described challenges in accessing care, two key factors help explain why these challenges were not prohibitive to this group initiating and completing treatment: provision of treatment at no cost, and readiness for treatment and expectations about healthcare access. Firstly, the fact that the treatment was provided at no cost to people in the program was extremely important, because private treatment was unaffordable for all participants. These interviews were conducted in 2018, when there was low availability of DAAs in Myanmar even in the private sector, and prices were extremely high. By 2020, prices had fallen dramatically with DAAs purchased at USD93 per course by the national program [4]. Prices in private sector are difficult to determine. Since then, DAAs are now available in the private sector or through non-government organisations (on a small scale); with DAAs available at no cost through some non-government organisations or at reduced prices. Many participants described how, prior to the national program roll-out, the cost of treatment was prohibitive, and resulted in delays in seeking care. The availability of free treatment outweighed other inconveniences and challenges in accessing care. Participants were willing to accept inconvenient models of care, long waiting times, extensive travel to reach care, and perceived lower quality of care if they could receive free treatment. Similar findings were reported in a study from Georgia, where participants interviewed were so grateful for the free treatment that they were willing to ‘tolerate everything’ to get treatment [33]. The introduction of costs might change the proportion of people willing to sacrifice time and suffer inconvenience for the benefit of treatment. Further work to determine how to appropriately deliver cost-sharing programs should be considered. Furthermore, our findings and those from Georgia [33] highlight that there are layers of the inequity in accessing healthcare. In these resource-constrained settings, this begins with the (often limited) availability of subsidised treatment through national programs and is further compounded by an individual’s financial capability and distance from healthcare. Future models of care for hepatitis C specifically designed for delivery in LMICs must account for these layered inequities and ensure that the right to access healthcare is upheld by reducing the difficulties in reaching and accessing with care.

Secondly, many participants believed that they should prioritise their own health and must overcome any difficulties to ensure they receive care; this indicates that these participants were ready to undertake treatment and willing to do what was required to take advantage of the opportunity for free treatment. Interlinked with this belief, there were widely held expectations surrounding how public and free healthcare is provided in Myanmar. In this setting, existing expectations surrounding how public healthcare is delivered in Myanmar, including reliance on walk-in appointment times and queuing, may have normalised wait times and helped to overcome traditional barriers to healthcare access among interviewees. Participants were mostly satisfied with the care received. Participants acknowledged that that the healthcare team was trying their best to provide care to the many people attending, but they would expect more if they were accessing care through the private system.

It is worth noting that there was some infidelity in how the program protocol was implemented, in that some people were erroneously charged for pre-treatment investigations. These participants typically reported paying USD5–10 for pre-treatment investigations. While these costs are quite low, they would still be a barrier to care for some treatment seekers. Quantitative evaluation data from the Myanmar national program published by Boeke et al. (2020) demonstrated that the major gap in the care cascade was between antibody testing and RNA testing (57%), while almost all of those with confirmed hepatitis C infection initiated on treatment (97%) [29]. It is unclear whether the gap in uptake of RNA testing is due to loss to follow-up or program capacity to provide treatment. If this gap is partly due to loss to follow-up, a contributing factor may have been the requirement to pay for pre-treatment investigations, because pre-treatment investigations would have occurred prior to RNA testing according to the protocol designed to reduce the number of unnecessary RNA tests (the most expensive component of pre-treatment assessments). When the results of the evaluation report were presented to the NHCP and consultant hepatologists, they were surprised to learn that some participants reported having paid for these pre-treatment investigations, because the national program had issued an official letter to all sites explaining that the program would cover these costs. This can easily be resolved in any future expanded roll-out, and confirmed through annual audits of patient experience at each site. The likely barrier of cost to starting treatment is supported by qualitative findings on acceptability and accessibility of a community-based hepatitis C testing and treatment program in Yangon, where participants described their reluctance to access treatment previously as they had been told by their networks of high treatment costs at both public and private health centres in Yangon and elsewhere in Myanmar [20].

Based on experiences in other settings, intersecting stigma and discrimination is a barrier to care for many people who inject drugs in Myanmar, and other marginalised groups who have previously experienced stigma and discrimination when accessing healthcare [11, 34, 35]. The participants we interviewed did not raise this as a major concern; however, we only interviewed those who successfully completed treatment, and few interviewees reported currently injecting drugs. A few participants mentioned their ‘bad lifestyle’ or blamed themselves for acquiring hepatitis C, indicating some internalised shame and stigma, which may have reduced their agency to ask questions of the healthcare staff.

Receipt of patient education within the national program differed between interviewees. Participants receiving personalised health advice, one-on-one consults with enough time to ask questions, and written information were more satisfied with their patient education and counselling, and those receiving written information appearing to retain pertinent information better. The influence of the power imbalance between patients and providers, and of cultural norms on acceptance of care, particularly when treatment is free, was observed in relation to not asking many questions of the healthcare team. Ensuring people are provided with their own individualised recommendations for any hepatocellular carcinoma surveillance and ongoing liver-related healthcare, including general advice to reduce alcohol intake and complete hepatitis B vaccination schedules, is essential to provision of high-quality, guideline-based care. Providing clear and accurate information on hepatitis C transmission routes to empower people to reduce their risk of reinfection is also important, particularly for people who inject drugs. In a Rwandan study, many participants did not understand how they had acquired hepatitis C or transmission routes, and had little understanding of liver health and care, with one patient wondering if their chest pain was related to their hepatitis C [17]. Similar issues surrounding lack of knowledge about transmission routes and symptoms are often reported as barriers to care [36, 37]. A recent study exploring ways to improve retention of hepatitis C knowledge among people who use drugs found that a short, story-telling video format was helpful for improving knowledge and retention of knowledge, compared to standard brochure format [38]. Provision of accurate and simple hepatitis C education resources, in written and short video format, will be important to improving quality of care within the program in Myanmar. In addition, standardised clinical training regarding hepatitis C transmission routes and filling the human resourcing gap to allow for longer consultations within which to discuss individuals’ situations will also be critical to improving quality of and satisfaction with care.

Scaled-up delivery and decentralisation of the program may overcome some of the structural barriers to accessing care, namely reducing the cost and time involved in transportation to sites, by increasing the coverage of sites across Myanmar. In addition to expansion of hospital sites, decentralisation of hepatitis C care should include provision at township medical centres or equivalent primary healthcare sites. For some, even minimal transport costs would prevent access to care, requiring specific interventions. For example, a United States study of people living with HIV attempting to access care found that reimbursement of transport costs was ineffective because many people could not afford the upfront payments, and recommended use of transport vouchers instead [39]. In other settings, including LMICs, trials of food and transport vouchers to cover cost of food and transport for attending care have shown promising results in improving attendance for HIV prevention procedures and care [40–42]. Financial barriers to reaching care due to transport costs, lost income, and the number of required appointments are commonly reported in studies of barriers to hepatitis C care in many settings, including high-income countries [15, 17, 18]. Even minimal transportation costs can be prohibitively expensive for those with little income and few financial assets. Some participants in the Rwandan study mentioned earlier relied on borrowing money from friends and family to cover the cost of transportation [17]. Task-shifting and decentralisation of care for hepatitis C is now supported by WHO Guidelines, with systematic reviews demonstrating the positive impact on access to care and retention in care [5, 43]. In Myanmar, a community-based, simplified model of care was trialled in Yangon for people who inject drugs and general population groups at two sites, with results supporting the feasibility of this care model and the positive impact on retention in care, with high rates of treatment uptake, completion and SVR [44, 45]. In addition to decentralising care locations, telemedicine could also assist in overcoming barriers to accessing care, such as distance to clinic, with initial evidence from HIV programs in LMICs showing telemedicine can relieve travel burden on patient [46].

Scaling up and decentralising the national hepatitis C treatment program in Myanmar in its current form would not necessarily solve the issues of wait time, number of appointments required, and insufficient consultation length and content. In the South African study mentioned earlier, waiting time for appointments was also highlighted as a barrier to care, particularly for those on methadone or facing drug withdrawal who could not arrive early in the morning to queue because they had to attend daily methadone dosing first or were not prepared to wait all day [18]. Introducing an appointment time or morning and afternoon sessions system could be helpful in reducing unnecessary wait times for people. However, this would likely increase costs, including for nurse staff time to call people or implementation of an automated electronic reminder system. Simplification of the clinical pathway and streamlining of visits to reduce the number of appointments people are required to attend could improve retention in care and reduce the workload of healthcare providers. As highlighted in the findings, each appointment required time off work and resulted in lost income for some participants. The CT2 Study in Myanmar and the Médecins Sans Frontières (MSF) program in Cambodia both demonstrated that simplified clinical pathways and streamlined service delivery were safe and effective, with high retention in care and good treatment outcomes [44, 47]. The Cambodia MSF program showed that iterative simplification of the clinical pathway reduced the costs of implementation but had no impact on effectiveness [47–49].

**Fig. 2:**
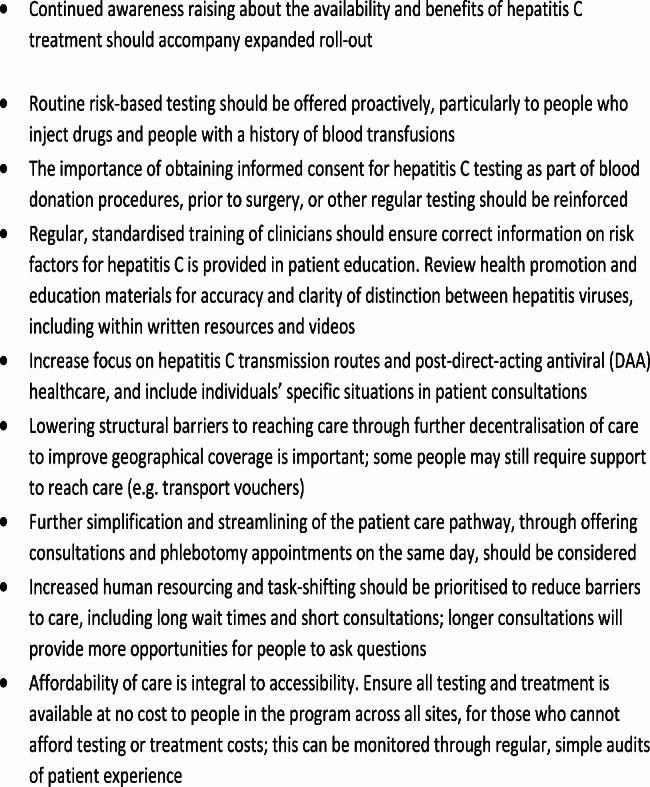
Considerations for service refinement & expanded roll-out

Task-shifting to predominately general practitioner-led care will increase the number of providers available to treat hepatitis C and ensure specialists are available to manage more complex cases [6]. Further task-shifting to nurses, particularly for patient education, should be considered. In this study, nurses were often praised and described as helpful and kind. While it was difficult to differentiate who delivered patient education at each site (nurse or doctor), task-shifting of patient education to nurses may allow more time to be spent with each patient to discuss topics of interest. Further, prior to further scale-up and decentralisation of the program, adequate clinical training covering risk factors, patient education approaches (particularly on how to convey information about the various hepatitis viruses), and referral criteria and pathways must be provided. Considerations for service refinements and expanded roll-out based on the study findings are summarised in Fig. 2.

### Strengths & limitations

This study contributes to filling the gap in the literature on people’s experience of hepatitis C treatment in LMICs. The findings provide useful evidence to inform continued program roll-out and refinement in Myanmar. A strength of this study is that we interviewed participants from all of the national program sites, identifying some issues (e.g. people having to pay for pre-treatment investigations) that would not have been captured if only recruiting from selected national program sites.

The major limitation of this qualitative evaluation of people’s experience of accessing care was that only those who successfully initiated and completed treatment were interviewed. Due to logistical constraints, we were unable to interview those who were not retained in care throughout. Therefore, the findings reflect the experiences of those who completed the care pathway only. As such, we were unable to identify barriers that prevented care – including those related to the approachability of the program – and that resulted in people failing to complete the care pathway. However, the barriers that participants overcame to access care provide insight into which aspects of the program may prevent others from obtaining care. These findings are likely transferable to other settings, with details of the specific context for assessment of transferability.

Another potential limitation of this work is social desirability bias; it is possible that the participants interviewed were unwilling to be critical of the national program due to receipt of free treatment or because interviews took place at the hospital. Also, the sampling strategy (convenience sampling of 4–5 participants per site) may mean that we did not capture all viewpoints. More viewpoints may have been captured if we employed a purposive/quota or judgement sampling strategy and ensured that we recruited participants from various key population groups or socio-economic situations.

## Conclusion

We identified a range of barriers to and enablers of access to hepatitis C care through the Myanmar national program during its first phase of roll-out, including structural barriers that might prevent many others from accessing care. These barriers should be overcome through expanded implementation across more geographical locations and increased human resourcing. Improving the care provided could also be achieved through greater focus on patient education and counselling content and modes of delivery, including standardised clinician education on hepatitis C transmission routes. Our results can be used to refine the care model, including through streamlining care pathway requirements for people and implementing an appointment-based model. Importantly, these results confirm that providing free treatment is critical to enabling many people with hepatitis C to access care. While expansion of Myanmar’s national hepatitis C treatment program has stalled due to the COVID-19 pandemic and the changed political landscape, continued provision of free treatment to those who cannot afford to pay should be prioritised in the next stage of elimination efforts.

### Electronic supplementary material

Below is the link to the electronic supplementary material.

Supplementary Material 1

## Data Availability

The qualitative interview dataset analysed for this study is not publicly available to protect the privacy of those interviewed but may be available from the corresponding author on reasonable request.

## List of abbreviations

CHAI: Clinton Health Access Initiative
DAA: direct-acting antivirals
LMICs: low and middle-income countries
MSF: Médecins Sans Frontières
NHCP: National Hepatitis Control Program
SVR: sustained virological response

## References

[1] Blach S, Terrault NA, Tacke F, Gamkrelidze I, Craxi A, Tanaka J (2022). Global change in Hepatitis C virus prevalence and cascade of care between 2015 and 2020: a modelling study. Lancet Gastroenterol Hepatol.

[2] World Health Organization (WHO). Global progress report on HIV, viral hepatitis and sexually transmitted Infections, 2021. Accountability for the global health sector strategies 2016–2021: actions for impact. Geneva; 2021.

[3] World Health Organization (WHO). Progress report on access to hepatitis C treatment: focus on overcoming barriers in low- and middle-income countries. Geneva; 2018.

[4] Clinton Health Access Initiative (CHAI). HCV Market Intelligence Report 2021 and Preliminary HBV Market Insights. 2021.

[5] World Health Organization (WHO). Updated recommendations on treatment of adolescents and children with chronic HCV Infection, and HCV simplified service delivery and diagnostic. Geneva; 2022. 37410875

[6] Draper B, Yee WL, Pedrana A, Kyi KP, Qureshi H, Htay H (2022). Reducing liver disease-related deaths in the Asia-Pacific: the important role of decentralised and non-specialist led Hepatitis C treatment for cirrhotic patients. Lancet Reg Health West Pac.

[7] World Health Organization (WHO). Accelerating access to Hepatitis C diagnostics and treatment: overcoming barriers in low and middle-income countries. Global progress report 2020. Geneva; 2021.

[8] Bajis S, Dore GJ, Hajarizadeh B, Cunningham EB, Maher L, Grebely J (2017). Interventions to enhance testing, linkage to care and treatment uptake for Hepatitis C virus Infection among people who inject Drugs: a systematic review. Int J Drug Policy.

[9] Bajis S, Applegate TL, Grebely J, Matthews GV, Dore GJ, Novel Hepatitic C, Virus (HCV) Diagnosis and Treatment Delivery Systems, editors. Facilitating HCV Elimination by Thinking Outside the Clinic. J Infect Dis. 2020;222(S9):S758–72. 10.1093/infdis/jiaa366 33245354

[10] Grebely J, Applegate TL, Cunningham P, Feld JJ (2017). Hepatitis C point-of-care diagnostics: in search of a single visit diagnosis. Expert Rev Mol Diagn.

[11] Amoako A, Ortiz-Paredes D, Engler K, Lebouché B, Klein MB (2021). Patient and provider perceived barriers and facilitators to direct acting antiviral Hepatitis C treatment among priority populations in high income countries: a knowledge synthesis. Int J Drug Policy.

[12] Gunn J, Draper, Pedrana A, Sacks-Davis R, Keefe O’, Crawford S et al. A mixed-methods systematic review of barriers and enablers to Hepatitis C Care among people who inject Drugs. In: INHSU. 2021.

[13] Degenhardt L, Webb P, Colledge-Frisby S, Ireland J, Wheeler A, Ottaviano S (2023). Epidemiology of injecting drug use, prevalence of injecting-related harm, and exposure to behavioural and environmental risks among people who inject Drugs: a systematic review. Lancet Glob Health.

[14] Nelson PK, Mathers BM, Cowie B, Hagan H, Des Jarlais D, Horyniak D (2011). Global epidemiology of Hepatitis B and Hepatitis C in people who inject Drugs: results of systematic reviews. Lancet.

[15] Sherbuk JE, Tabackman A, McManus KA, Kemp Knick T, Schexnayder J, Flickinger TE (2020). A qualitative study of perceived barriers to Hepatitis C care among people who did not attend appointments in the non-urban US South. Harm Reduct J.

[16] Litwin AH, Drolet M, Nwankwo C, Torrens M, Kastelic A, Walcher S (2019). Perceived barriers related to testing, management and treatment of HCV Infection among physicians prescribing opioid agonist therapy: the C-SCOPE study. J Viral Hepat.

[17] Serumondo J, Penkunas MJ, Niyikora J, Ngwije A, Kiromera A, Musabeyezu E et al. Patient and healthcare provider experiences of Hepatitis C treatment with direct-acting antivirals in Rwanda: a qualitative exploration of barriers and facilitators. BMC Public Health. 2020;20(1). 10.1186/s12889-020-09000-0 PMC7298738 32546216

[18] Versfeld A, Versfeld A, McBride A, McBride A, Scheibe A, Scheibe A (2020). Motivations, facilitators and barriers to accessing Hepatitis C treatment among people who inject Drugs in two South African cities. Harm Reduct J.

[19] Le Nguyen M, Nguyen Thi Hong Y, Dang Trong T, Dung NT, Day J, Phuong LT (2022). Balancing uncertainty and proactivity in care seeking for Hepatitis C: qualitative research with participants enrolled in a treatment trial in Ho Chi Minh City, Vietnam. Int J Qual Stud Health Well-being.

[20] Yee WL, Bowring A, Draper B, O’Keefe D, Htay H, Myint KT (2023). Patients’ access to and acceptance of community-based Hepatitis C testing and treatment in Myanmar: a mixed-method study. PLOS Glob Public Health.

[21] Lwin AA, Aye KS, Moh Htun M, Kyaw YY, Ko Zaw K, Aung TT (2017). Sero-prevalence of Hepatitis B and C viral Infections in Myanmar: National and Regional Survey in 2015. Myanmar Heal Sci Res J.

[22] Johnston LG, Soe P-M, Aung MY, Ammassari S (2019). Estimating the Population size of males who inject Drugs in Myanmar: methods for obtaining Township and National estimates. AIDS Behav.

[23] Myanmar National Hepatitis Control Program. Myanmar National Strategic Plan on Viral Hepatitis 2016–2020. 2017.

[24] Myanmar National Hepatitis Control Program. Myanmar National Action Plan for Viral Hepatitis Response 2017–2020. 2017.

[25] Ministry of Health and Sports Myanmar. Myanmar National Simplified Treatment Guidelines for Hepatitis C Infection. 2017.

[26] Ministry of Health and Sports Myanmar. Myanmar National Simplified Treatment Guidelines for Hepatitis C Infection - Second Edition, July 2019. 2019.

[27] Min Thaung Y, Chasela CS, Chew KW, Minior T, Lwin AA, Sein YY (2021). Treatment outcomes and costs of a simplified antiviral treatment strategy for Hepatitis C among monoinfected and HIV and/or Hepatitis B virus-co‐infected patients in Myanmar. J Viral Hepat.

[28] Myanmar National AIDS. Program. Myanmar Integrated Biological and Behavioural Survey & Population Size Estimates among People Who Inject Drugs (PWID) 2017–2018. 2019.

[29] Boeke CE, Adesigbin C, Agwuocha C, Anartati A, Aung HT, Aung KS (2020). Initial success from a public health approach to Hepatitis C testing, treatment and cure in seven countries: the road to elimination. BMJ Glob Heal.

[30] Neale J (2016). Iterative categorization (IC): a systematic technique for analysing qualitative data. Addiction.

[31] Levesque J-F, Harris MF, Russell G (2013). Patient-centred access to health care: conceptualising access at the interface of health systems and populations. Int J Equity Health.

[32] Myanmar raises minimum. wage to $4.80 a day as economy staggers [Internet]. The Straits Times. 2018 [cited 2022 Mar 11]. Available from: https://www.straitstimes.com/asia/se-asia/myanmar-raises-minimum-wage-to-s480-a-day-as-economy-staggers.

[33] Chikovani I, Ompad DC, Uchaneishvili M, Sulaberidze L, Sikharulidze K, Hagan H (2019). On the way to Hepatitis C elimination in the Republic of Georgia—barriers and facilitators for people who inject Drugs for engaging in the treatment program: a formative qualitative study. PLoS ONE.

[34] Skeer MR, Ladin K, Wilkins LE, Landy DM, Stopka TJ (2018). Hep C’s like the Common Cold’: understanding barriers along the HCV care continuum among young people who inject Drugs. Drug Alcohol Depend.

[35] Madden A, Hopwood M, Neale J, Treloar C (2018). Beyond interferon side effects: what residual barriers exist to DAA Hepatitis C treatment for people who inject Drugs?. PLoS ONE.

[36] McGowan CE, Fried MW (2012). Barriers to Hepatitis C treatment. Liver Int.

[37] Khaw F-M, Stobbart L, Murtagh MJ (2007). I just keep thinking I haven’t got it because I’m not yellow: a qualitative study of the factors that influence the uptake of Hepatitis C testing by prisoners. BMC Public Health.

[38] Talal A, Ding Y-X, Markatou M (2022). Innovations in education: a prospective study of storytelling narratives to enhance Hepatitis C virus knowledge among substance users. World J Hepatol.

[39] Sagrestano LM, Clay J, Finerman R, Gooch J, Rapino M (2014). Transportation vulnerability as a barrier to service utilization for HIV-positive individuals. AIDS Care.

[40] Kennedy CE, Yeh PT, Atkins K, Fonner VA, Sweat MD, O’Reilly KR et al. Economic compensation interventions to increase uptake of voluntary medical male circumcision for HIV prevention: A systematic review and meta-analysis. Francis JM, editor. PLoS One. 2020;15(1):e0227623. 10.1371/journal.pone.0227623 PMC6961886 31940422

[41] Thirumurthy H, Masters SH, Rao S, Bronson MA, Lanham M, Omanga E (2014). Effect of providing conditional economic compensation on Uptake of Voluntary Medical Male Circumcision in Kenya. JAMA.

[42] Govindasamy D, Ford N, Kranzer K (2012). Risk factors, barriers and facilitators for linkage to antiretroviral therapy care. AIDS.

[43] Oru E, Trickey A, Shirali R, Kanters S, Easterbrook P (2021). Decentralisation, integration, and task-shifting in Hepatitis C virus Infection testing and treatment: a global systematic review and meta-analysis. Lancet Glob Health.

[44] Draper BL, Htay H, Pedrana A, Yee WL, Howell J, Pyone Kyi K (2021). Outcomes of the CT2 study: a ‘one-stop‐shop’ for community‐based Hepatitis C testing and treatment in Yangon, Myanmar. Liver Int.

[45] Draper BL, Yee WL, Shilton S, Bowring A, Htay H, Nwe N (2022). Feasibility of decentralised, task-shifted Hepatitis C testing and treatment services in urban Myanmar: implications for scale-up. BMJ Open.

[46] Phan JM, Kim S, Linh ĐTT, Cosimi LA, Pollack TM (2022). Telehealth interventions for HIV in Low- and Middle-Income Countries. Curr HIV/AIDS Rep.

[47] Zhang M, O’Keefe D, Craig J, Samley K, Bunreth V, Jolivet P (2021). Decentralised Hepatitis C testing and treatment in rural Cambodia: evaluation of a simplified service model integrated in an existing public health system. Lancet Gastroenterol Hepatol.

[48] Walker JG, Mafirakureva N, Iwamoto M, Campbell L, Kim CS, Hastings RA (2020). Cost and cost-effectiveness of a simplified treatment model with direct‐acting antivirals for chronic Hepatitis C in Cambodia. Liver Int.

[49] Zhang M, O’Keefe D, Iwamoto M, Sann K, Kien A, Hang V (2020). High sustained viral response rate in patients with Hepatitis C using generic sofosbuvir and daclatasvir in Phnom Penh, Cambodia. J Viral Hepat.

