# Supplementary material for: Patients’ experience of accessing hepatitis C treatment through the Myanmar national hepatitis C treatment program: a qualitative evaluation
Source: BMC Health Serv Res. 2024 Jan 16;24:80. doi: 10.1186/s12913-023-10456-0 (PMC10792827; doi:10.1186/s12913-023-10456-0)
Supplement: Supplementary file 1 — Supplementary Material 1 [file 12913_2023_10456_MOESM1_ESM.pdf]

Supplementary Material 1. Participant Interview Guide

It is worth noting that depending on the interviewee's responses some questions in the interview guide may not be asked. Alternatively, further details may be sought following some questions with interviewees asked to expand on their answers. However, any additional questions will be directly relevant to the QuickStart program.

1. Demographics & Previous Healthcare Utilization

1.1 Age
1.2 Gender
1.3 Township (Ward) of residence
1.4 Estimated years of hepatitis C infection
1.5 Estimated years since first diagnosis of hepatitis C infection
1.6 How do you think you got hepatitis C?

(*Prompts: Blood transfusion, Renal dialysis, Surgery, Dental treatment, Tattooing/scarification, Injecting drug use, Unsafe sex, Family member with chronic hepatitis C infection, Unknown)*

1.7 Have you ever: *(choose all that apply)*

a) Been admitted to hospital
b) Had a blood transfusion (e.g. get given blood over a few minutes or more)
c) Had surgery
d) Had injections (including vaccinations, medications – NOT BLOOD)
e) Had stitches
f) Had an endoscopy (Medical investigation of your internal organs using a thin tube with a camera)
g) Been given fluids/salty water or medicines through a needle or tube in your arm?
h) Had a catheter (a tube placed inside your urethra to let out urine)
i) Had gum treatment
j) Had teeth extractions
k) Given birth
l) Had a caesarean section
m) Injected drugs
n) Had renal dialysis
o) Had a tattoo/scarification
p) Had sex with a HCV positive person
q) Had a family member with chronic HCV infection
r) Had a family member with liver disease

1.8 Have you ever been to a: *(choose all that apply)*

a) Quack
b) Traditional medicine clinic
c) Public clinic
d) Midwife, even if not pregnant/giving birth
e) Maternal Child Health centre
f) Private generalist doctor
g) Private clinic
h) INGO clinic
i) Pharmacy
j) Sub Rural Health Centre (RHC)
k) Rural Health Centre (RHC)
l) Station hospital

m) Township hospital
n) Divisional hospital
o) Specialty hospital
p) Other medical service: ___________________________________

2. Patient journey description
2.1 Current stage of hepatitis C treatment journey

- ☐ Started on treatment
- ☐ Completed treatment
- ☐ Obtained SVR12 result – cured
- ☐ Obtained SVR12 result – not cured

2.2 How did you first attend [site] for hepatitis C screening/treatment?
2.2.1 Had you been to the site before?
2.2.2 Were you referred by another service?

- How did that work?
- How long did you wait between getting referred to the site and having your first appointment?

2.2.3 How did you get to the site?

- How long did it take each way?
- How much did it cost each way?
- Did you need to pay for food or accommodation or time off work to attend appointment? If so, how much for each?

2.3.4 How long did you wait for your first appointment?
2.3 Thinking about the hepatitis C screening test (antibody test):
2.3.1 How acceptable was this test to you? Why was it acceptable/not acceptable?
2.3.2 How long did it take you to receive this test result from when you got the blood sample taken?

2.3.2.1 Would you prefer to get your hepatitis C test result on the same day as getting tested?

2.3.3 Did you have to do anything with the blood sample after it was taken?

- Take it to the laboratory?
- Pay for the test to be performed?
- Pick up result from the laboratory?

2.3.4 How did you receive the result?

- From who
- Did you receive the test result on the same day as the sample was taken

2.3.5 Did you need to pay to have test done or to receive result?
2.4 Thinking about the hepatitis C confirmatory test (PCR/RNA test):

2.4.1 How acceptable was this test to you? Why was it acceptable/not acceptable?2.4.2 How long did it take you to receive this test result from when you got the blood sample taken?

2.4.2.1 Would you prefer to get your hepatitis C test result on the same day as getting tested?

2.4.3 Did you have to do anything with the blood sample after it was taken?

- Take it to the laboratory?
- Pay for the test to be performed?
- Pick up result from the laboratory?

2.4.4 How did you receive the result?

- From who
- At what appointment

2.4.5 Did you need to pay to have test done or to receive result?

2.5 Thinking about when you started hepatitis C treatment,
2.5.1 Did you have a blood test to assess your liver health?
2.5.2 Are you confident that you understood the result of the blood test?
2.5.3 Did you have a scan of your liver? (If not done/available, skip 2.5.4)
2.5.4 Are you confident that you understood the result of the scan?
2.5.5 Did you receive the results of your liver health tests at the same appointment as when you started on treatment?
2.6 Thinking about when you started hepatitis C treatment,
2.6.1 Did you understand your treatment plan?
2.6.2 How often did you need to attend the clinic to pick up your treatment medication?
2.6.3 Did you need to pay anything to pick up the treatment medication?
2.6.4 Did you see the doctor when you picked up the treatment medication?
2.6.5 How long after your first appointment did you start treatment?
2.6.6 How did you know when to attend your next appointment?
2.7 While you were on treatment;
2.7.1 Did you experience any side effects?
2.7.2 Did you stop treatment due to side effects?
2.7.3 Did you miss any doses? If yes, how many doses did you miss?
2.8 Thinking about when you had your ‘cure’ blood test (12 weeks after you finished treatment);
2.8.1 How did you know when to get the blood test done?
2.8.2 Where did you go to get the blood test done?
2.8.3 When did you get the result of the test?
2.8.4 Are you confident that you understand the result of this blood test?
2.8.5 Are you confident that you understand what you need to do to make sure you don’t get hepatitis C again?
2.8.6 Are you confident that you understand how to look after your liver now?
Thinking back to before you started treatment and comparing to now:
2.9.1 How do you feel now that you have been treated?

2.9.2 How has being treated changed things for you?
2.9.3 How are you feeling physically and mentally? Has anything changed socially for you?
2.9.4 Has completing treatment and being clear of hepatitis C motivated you to do other things or act any differently?
(Prompts: with your health behaviours, drug use or interacting with family and friends)

3. Satisfaction

3.1 Thinking about your hepatitis C care at [site], how satisfied were you with:
(Please note down any reasons why)
3.1.1 How the HCV testing process was explained to you
3.1.2 How the test samples were taken
3.1.3 How the test results were explained to you
3.1.4 How the treatment process was explained to you
3.1.5 How often you had to attend the clinic
3.1.6 The medical and nursing support available to you whilst on treatment
3.1.7 The overall hepatitis C care you have received

3.2 Thinking about your hepatitis C care at [site], how much do you agree or disagree with these statements?
(Please note down any reasons why)
3.2.1I trust that the staff at [site] are well-trained and can provide quality healthcare for hepatitis C
3.2.2 I am confident that the test results the staff provided to me are correct
3.2.3 I am confident that the treatment plan the staff put me on was the best for me
3.2.4 I felt comfortable telling the staff about my behaviours that might put me at risk of hepatitis C
3.2.5 I felt confident that my medical information was securely stored
3.2.6 I felt confident that my privacy was respected
3.2.7 I had enough information given to me before starting treatment
3.2.8 If I asked for specific information, the doctors gave it to me

3.3 Thinking about your hepatitis C care at [site], how much do you agree or disagree with these statements?
(Please note down any reasons why)
3.3.1 I found the location of the clinic was convenient to me
3.3.2 The wait time at the clinic was reasonable
3.3.3 There was no delay in starting treatment
3.3.4 The clinic rooms were clean
3.3.5 The clinic rooms were private enough for the consultations
3.3.6 The staff were friendly
3.3.7 I would recommend this clinic to my friends/family

Thank you for your time
